# Knowledge, Attitudes and Practices (KAP) on Dengue Fever in Burkina Faso: Findings from the national household survey in Burkina Faso

**DOI:** 10.1371/journal.pntd.0014027

**Published:** 2026-08-31

**Authors:** Nicolas Ouédraogo, Siaka Débé, Harouna Soré, Farida Tiendrébégo, Gérard Wendyam Nonkani, Guillaume Sylvestre Sanou, Réné Kinda, Adama Ganou, Casimire Wendlamita Tarama, Sonia Ilboudo, Moussa Wamdaogo Guelbéogo, Isaie Medah, Adama Gansané

**Affiliations:** 1 Centre National de Recherche et de Formation sur le Paludisme, Institut National de Santé Publique, Ouagadougou, Burkina Faso; 2 Université Joseph Ki Zerbo, Ouagadougou, Burkina Faso; 3 Direction Scientifique, Institut National de Santé Publique, Ouagadougou, Burkina Faso; Kwame Nkrumah University of Science and Technology, GHANA

## Abstract

**Background:**

Dengue fever is an emerging public health concern in Burkina Faso, with increasing outbreaks and data gaps in population awareness. This study assessed knowledge, attitudes, and practices (KAP) related to dengue fever in all health regions of the country.

**Methodology/principal findings:**

A nationwide cross-sectional household survey was conducted in May 2022 using a stratified two-stage cluster sampling design. One rural and one urban area were selected per region. Heads of households or their representatives were interviewed using a structured questionnaire. Data were collected electronically and analyzed using Stata. A total of 1,568 participants were enrolled (52.0% male; 48.0% female). Overall, 66.3% had heard of dengue, with higher awareness in urban than rural areas. Only 49.0% correctly identified mosquito bites as the mode of transmission, and 29.9% did not know what dengue is. Most respondents (88.3%) stated that dengue can affect everyone. Regarding prevention, 80.2% reported sleeping under a mosquito net, 49.0% eliminated stagnant water, and 45.4% used mosquito repellents. In practice, 67.6% consistently slept under mosquito nets and 82.6% used repellents. Almost all respondents (98.6%) reported that they would consult a health professional if they had symptoms of dengue. However, knowledge about treatment and vaccination was limited, with 46.5% and 56.6% respectively reporting not knowing whether drugs or vaccines exist.

**Conclusions/significance:**

This study highlights moderate awareness but substantial knowledge gaps and urban–rural disparities in dengue-related KAP in Burkina Faso. Strengthening community-based education and integrated vector control strategies is essential to improve prevention and reduce dengue transmission.

## Author summary

Dengue fever is a mosquito-borne disease that is spreading in many parts of the world, including West Africa. In Burkina Faso, outbreaks have been reported in recent years, but little is known about what communities understand about the disease or how they protect themselves. In this study, we conducted a nationwide household survey covering all health regions of the country to explore people’s knowledge, perceptions, and prevention practices related to dengue. We found that while many people had heard about dengue, important gaps remain. A significant proportion did not know how the disease is transmitted or whether treatments or vaccines exist. Preventive actions such as sleeping under mosquito nets and removing standing water were reported, but these practices were not consistent everywhere, especially between urban and rural areas. Encouragingly, almost all respondents said they would seek care from a health professional if they developed symptoms. Our work provides the first national picture of community awareness and behaviors related to dengue in Burkina Faso. These findings highlight the need for strengthened health education and community engagement to improve prevention and support ongoing efforts to control mosquito-borne diseases.

## Introduction

Dengue fever (DF) is a primary viral disease transmitted to humans by mosquitoes (*Aedes aegypti et Aedes albopictus)*, imposing a significant economic and health burden in numerous regions globally [1–3]. The global incidence of dengue has markedly increased over the past two decades, posing a substantial public health challenge. From 2000 to 2019, the World Health Organization documented a ten-fold surge in reported cases worldwide, increasing from 500,000 to 5.2 million. The year 2019 marked an unprecedented peak, with reported instances spreading across 129 countries. In 2023, dengue fever continued to pose a major public health challenge worldwide, with a substantial increase in cases reported in several endemic regions, including Burkina Faso, a country already facing a difficult security situation [4]. The first cases of dengue fever have been reported in Burkina Faso as early as 1925 and again in 1980 [5]. Larger outbreaks occurred in 2016 and 2017, leading to the strengthening of surveillance system in the country through the establishment of sentinel sites [6]**.** In 2016, the country recorded 2,600 cases and 21 deaths, followed by 14,944 cases and 30 deaths in 2017.

In 2023, the country becomes the most affected in the African region, experiencing a significant increase in dengue cases compared with the same periods in 2021 and 2022. Outbreak in 2023 showed higher trends in mortality and morbidity compared to those seen in 2016 and 2017 with a cumulative number of cases reported in the country at 146,878 suspected cases, including 68,346 probable cases (positive rapid diagnostic test) and 688 deaths among suspected cases, representing a case fatality rate of 0.5% [4,7].

Despite efforts by the Ministry of Health to strengthen dengue surveillance and case management, self-medication and the use of traditional remedies remain common in many communities during febrile illnesses, reflecting the continued reliance on traditional medicine in Burkina Faso [8,9]. In addition, dengue fever is frequently confused with malaria because both diseases share similar clinical manifestations, including fever, headache, and body pain, which may delay appropriate diagnosis and treatment [10,11]. This misidentification leads to delays in seeking appropriate medical care and contributes to the spread and severity of the disease. Furthermore, the proliferation of unverified treatment methods and misinformation about the disease complicates public health efforts to control the outbreak [12].

Understanding the knowledge, attitudes, and practices (KAP) of the population regarding dengue fever is crucial for designing effective public health interventions. Assessing the level of awareness and behavior of the population will help identify gaps in knowledge and guide the development of targeted strategies for dengue prevention and treatment. The aim of this study was to assess the population’s knowledge, attitudes and practices relating to dengue fever in the 13 health regions of Burkina Faso by means of a survey of the population (urban and rural).

## Methods

### Ethics statement

The project protocol received approval from the National Ethics Committee prior to the implementation of project activities (Reference No. 2020-9-195, dated September 2, 2020). Authorization was also obtained from the administrative, health, and security authorities in each region. Written informed consent was obtained from household heads or their authorized representatives prior to household-level data collection, and from each individual participant before personal data collection. As all study participants were heads of households or their legal representatives and were adults, no assent or parental/guardian consent was required. For participants who were unable to read or write, the consent form was read aloud in their preferred local language in the presence of an impartial witness, and consent was documented using a thumbprint in accordance with ethical committee recommendations. Participation was entirely voluntary, and respondents were informed of their right to decline participation or withdraw at any stage without any consequences. No financial compensation or incentives were provided to participants. However, as this KAP survey was nested within a prospective national dengue cohort, participants were entitled to free consultation and treatment for suspected dengue and malaria throughout the one-year follow-up period upon presentation of their study identification card at designated health facilities.These procedures ensured full compliance with ethical standards for research involving human participants.

### Study sites

The study was conducted nationwide across all 13 regions of Burkina Faso. One urban and one rural locality were selected from each region, resulting in a total of 26 study sites. The study sites are described in S1 Fig.

### Participant selection method

The present KAP survey was nested within a nationally representative dengue seroprevalence survey. Therefore, the sample size was determined according to the requirements of the primary serological study. A sampling plan combining stratified and cluster sampling was used. Stratification was based on the country’s 13 regions, and in each of these regions on the rural (peri-urban) and urban areas of each capital. Cluster sampling was based on the sampling frame available from the 2019 general population and housing census (GPHC 2019), and the sample was selected at two levels: enumeration areas and households. EAs are geographically delimited administrative census units defined during the GPHC 2019 by the national statistical authorities, each generally comprising approximately 200–250 households for census and survey purposes.

#### First stage: Choice of enumeration area.

The enumeration areas (EA) of the GPHC were used as clusters. Each EA comprises at least 250 households. In each stratum, 01 EA was selected. This choice was made by simple random selection.

#### Second stage: Selection of households.

Households were selected within each chosen enumeration area (EA) using systematic random sampling until the predetermined sample size allocated to that cluster was reached. When a selected household was found to be ineligible, it was replaced by the next household on the waiting list in chronological order.

#### Selection and administration of the participant questionnaire in households.

In each selected household, the household head or an adult representative (aged ≥18 years) present at the time of the survey was invited to participate in the cross-sectional study using the questionnaire following the obtention of the informed consented. Households were considered eligible if at least one adult respondent was available and willing to participate. Non-eligible households included vacant or non-residential dwellings, as well as households where no eligible adult respondent was available after repeated visits. When a selected household was unavailable or refused participation, the next household on the pre-established replacement list was approached. Refusal rates were very low during the survey.

#### Questionnaire development, adaptation, and data collection.

The questionnaire consisted of two sections: the first collected participants’ socio-demographic characteristics, while the second assessed their knowledge, attitudes, and practices (KAP) related to dengue. The KAP survey was conducted in selected households as part of a cross-sectional screening study in May 2022. Data were collected using a questionnaire adapted from previously published and validated dengue KAP survey tools and other vector-borne diseases, including WHO guidelines and validated community-based studies [30–32]. The instrument was developed following a review of published KAP studies and questionnaires obtained from their authors, and was subsequently adapted to the Burkina Faso context through expert review and contextual modifications related to local language, healthcare-seeking behavior, and dengue prevention practices. Prior to implementation, the questionnaire was pretested to assess clarity, relevance, and comprehensibility of the items. Information was collected through individual interviews conducted with the household head or a designated adult representative. The questionnaire was administered in French or, when necessary, translated into the participant’s local language with the assistance of a trained local interpreter, who also served as a guide to facilitate household access.

Prior to implementation, the questionnaire was pretested after the training of data collectors. Investigators were divided into two groups, and each group conducted a pilot administration of the questionnaire, considered as a pretest site. The pretest assessed the clarity, relevance, and comprehensibility of questions, their alignment with study objectives, the duration of administration, and the feasibility of household identification and localization procedures. Feedback collected during this exercise was reviewed by the study team, and minor adjustments were made to improve the wording, sequence, and contextual adaptation of the questionnaire before the survey implementation.

#### Data management and analysis.

Data were exported from the electronic data capture system into Microsoft Excel and analyzed using Stata/MP version 16.0 (StataCorp, College Station, Texas 77845 USA). Data cleaning procedures included consistency checks, management of missing values, and recoding of variables where necessary. Descriptive statistics were used to summarize sociodemographic characteristics and KAP variables using frequencies and percentages. Comparative analyses between rural and urban populations were performed using Pearson’s Chi-square test or Fisher’s exact test when appropriate. Statistical significance was set at p < 0.05. Graphical visualizations including radar charts and study maps were produced using DIVA-GIS software.

## Results

This study, conducted across all 13 health regions of Burkina Faso with a balanced inclusion of urban and rural populations, provides critical insights into the knowledge, attitudes, and practices (KAP) of the population regarding dengue fever. The findings reveal both promising awareness levels and significant gaps that warrant targeted public health interventions.

### Socio-demographic patterns

A total of 1,601 households were identified and approached for participation across 26 study sites nationwide. In this study, a “site” referred to a selected urban or rural locality within a health region. Accordingly, one urban and one rural locality were selected in each of the 13 health regions of Burkina Faso to ensure balanced geographic and demographic representation. The survey targeted household heads or, in their absence, an adult representative meeting the study eligibility criteria. Of the households approached, 1,568 respondents agreed to participate and were enrolled in the study, resulting in a participation rate of 97.9% and a refusal rate of 2.1%. The distribution of participants by region and site is presented in S2 Fig.

After obtaining informed consent from the participants, their socio-demographic characteristics were collected. These characteristics are presented in Table 1. Among the 1,568 respondents, 52.04% were male and 47.96% female, with a nearly equal distribution between rural and urban areas. The most represented age groups were 35–44 years (24.11%), 25–34 years (21.17%), and 45–54 years (20.15%). Most participants were married in monogamy (57.33%) or polygamy (23.22%). Over half (54.70%) had no formal schooling, and farming was the main occupation (38.26%), followed by trading (13.31%) and unemployment (12.41%).

**Table 1:** Socio-Demographic characteristics of study population (n = 1568).

Variables	Respondents distribution					
	Rural		Urban		Total	
	n = 782	%	n = 786	%	n = 1568	%
**Sex:**						
Male	413	26.34	403	25.7	816	52.04
Female	369	23.53	383	24.43	752	47.96
**Age:**						
15-24	77	4.91	84	5.36	161	10.27
25-34	146	9.31	186	11.86	332	21.17
35-44	185	11.80	193	12.31	378	24.11
45-54	168	10.71	148	9.44	316	20.15
55-64	115	7.33	80	5.10	195	12.44
65-74	60	3.83	65	4.15	125	7.97
75-84	26	1.66	28	1.79	54	3.44
85-94	3	0.19	2	0.13	5	0.32
95+	2	0.13	0	0.00	2	0.13
**Marital satatus:**						
Single	42	2.69	102	6.53	144	9.21
Divorcee	0	0	2	0.13	2	0.13
Married in monogamy	412	26.36	484	30.97	896	57.33
Married in polygamy	242	15.48	121	7.74	363	23.22
Widow/widower	8	5.12	70	4.48	150	9.6
NA	2	0.13	6	0.38	8	0.51
**Level of education:**						
Literacy	27	1.73	99	6.33	126	8.06
No schooling	541	34.61	314	20.09	855	54.7
Primary level	85	5.44	98	6.27	183	11.71
Secondary level	54	3.45	197	12.6	251	16.06
University level	2	0.13	76	4.86	78	4.99
NA	69	4.41	1	0.06	70	4.48
**Occupation:**						
Trader	27	1.73	181	11.58	208	13.31
Farmer	503	32.18	95	6.08	595	38.26
Breeder	36	2.3	6	0.38	42	2.69
Housewife	96	6.15	12	0.77	108	6.92
Pupil/ Student	19	1.22	52	3.33	71	4.54
Unemployed	51	3.26	143	9.15	194	12.41
Private sector	14	0.9	87	5.57	101	6.46
Retired	1	0.06	18	1.15	19	1.22
Public sector	3	0.19	109	6.97	112	7.17
Other	11	0.7	80	5.12	91	5.82
NA	17	1.09	2	0.13	19	1.22

This table presents the distribution of study participants by sex, age group, marital status, level of education, and occupation according to place of residence (rural and urban) in the national Knowledge, Attitudes and Practices (KAP) survey on dengue fever conducted in Burkina Faso.

### Knowledge of Dengue fever

To assess the KAP related to dengue fever, participants were first asked a general question on awareness of the disease (“Are you aware of the existence of a disease called Dengue”). Responses to this question are presented in Table 2. This initial step allowed the identification of respondents who reported awareness of dengue, among whom the subsequent analyses were conducted.

**Table 2:** Awareness of dengue fever among study participants according to place of residence in Burkina Faso (n = 1,568).

Response	Rural (n, %)	Urban (n, %)	Total (n, %)
Yes	419 (26.72%)	621 (39.60%)	1,040 (66.33%)
No	363 (23.15%)	165 (10.52%)	528 (33.67%)
Total	782 (49.87%)	786 (50.13%)	1,568 (100%)

This table shows participants’ responses regarding awareness of dengue fever, stratified by place of residence (rural and urban) in the national KAP survey conducted in Burkina Faso.

Analysis of household sources of information on dengue fever in Burkina Faso reveals a diversity of channels used by the population to access information. Knowledge of these sources is vital if we are to raise awareness and combat dengue fever more effectively. S3 Fig is a radar diagram illustrating the proportions of information channels on dengue fever used by participants in the cross-sectional survey of the present study.

Table 3 provides insights into participants’ knowledge regarding symptoms of Dengue fever virus infection. Overall, 49.04% of respondents identified dengue as a disease transmitted through mosquito bites, while 14.52% described it as an infectious disease and 29.91% reported that they did not know what dengue is. Only 1.44% mentioned that dengue is caused by virus-like microbes, and 0.29% correctly combined all three aspects (infectious, viral, and mosquito-borne).

**Table 3:** Knowledge of dengue fever among study participants in rural and urban settings in Burkina Faso (n = 1,568).

What is dengue fever?	Rural (n, %)	Urban (n, %)	Total	Percentage
1. Infectious disease	85 (8.17)	66 (6.35)	151	14.52
2. Caused by virus-like microbes	5 (0.48)	10 (0.96)	15	1.44
3. Transmitted through mosquito bite	175 (16.83)	335 (32.21)	510	49.04
4. Infectious disease + Caused by virus-like microbes	1 (0.10)	1 (0.10)	2	0.19
5. Infectious disease + Transmitted through mosquito bite	5 (0.48)	30 (2.88)	35	3.37
6. Caused by virus-like microbes + Transmitted through mosquito bite	1 (0.10)	12 (1.15)	13	1.25
7. Infectious disease + Caused by virus-like microbes + Transmitted through mosquito bite	0	3 (0.29)	3	0.29
8. Don’t know	147 (14.13)	164 (15.77)	311	29.91
Who gets dengue fever?				
1. Adults only	4 (0.38)	2 (0.19)	6	0.58
2. Children only	6 (0.58)	2 (0.19)	8	0.77
3. Poor people only	2 (0.19)	0	2	0.19
4. Rich people only	4 (0.38)	1 (0.10)	5	0.48
5. Elderly people only	2 (0.19)	5 (0.48)	7	0.67
6. Everyone	363 (34.90)	555 (53.37)	918	88.27
7. Don’t know	38 (3.65)	56 (5.38)	94	9.04
Is there a drug to treat the disease?				
1. Yes	62 (5.96)	172 (16.54)	234	22.50
2. No	161 (15.48)	161 (15.48)	322	30.96
3. Don’t know	196 (18.85)	288 (27.69)	484	46.54
Is there a vaccine to prevent dengue fever?				
1. Yes	16 (6.81)	30 (12.77)	46	19.57
2. No	15 (6.38)	41 (17.45)	56	23.83
3. Don’t know	31 (13.19)	102 (43.40)	133	56.60
Can the disease be repeated?				
1. Yes	268 (25.77)	318 (30.58)	586	56.35
2. No	22 (2.12)	31 (2.98)	53	5.10
3. Don’t know	129 (12.40)	272 (26.15)	401	38.56

This table presents participants’ knowledge of dengue fever, including perceptions of its nature, transmission, affected populations, treatment, vaccination, and reinfection, stratified by place of residence (rural and urban) in the national KAP survey conducted in Burkina Faso

Regarding populations affected, 88.27% stated that dengue can affect everyone, whereas 9.04% did not know. Very small proportions indicated that dengue affects only adults (0.58%), children (0.77%), the poor (0.19%), the rich (0.48%), or the elderly (0.67%).

Concerning treatment, 22.50% of respondents reported that a drug exists to treat dengue, 30.96% said no, and 46.54% did not know.

With respect to prevention, 19.57% reported the existence of a vaccine against dengue, 23.83% stated that there is no vaccine, and 56.60% did not know.

Finally, 56.35% indicated that dengue can be repeated, 5.10% said it cannot be repeated, and 38.56% did not know.

### Attitudes towards Dengue prevention and treatment

Dengue-related behaviours among participants, encompassing personal protection, self-treatment, and home care for relatives are listed in Table 4. Regarding personal protection, 80.19% of respondents reported sleeping under a mosquito net, 49.04% reported eliminating stagnant water, and 45.38% reported using mosquito repellent. Only 22.21% reported wearing long, loose clothing, while 7.12% stated that they do nothing to protect themselves from dengue. Other preventive actions such as cleaning the living environment were rarely mentioned (0.48%).

**Table 4:** Attitudes towards Dengue Fever.

What do you do to protect yourself from dengue fever?	Rural (n, %)	Urban(n, %)	Total	Percentage
1. Wear long, loose clothing^a^				
No	283 (27.21)	526 (50.58)	809	77.79
Yes	136 (13.08)	95(9.13)	231	22.21
2. Use mosquito repellent				
No	216(20.77)	352(33.85)	568	54.62
Yes	203(19.52)	269(25.87)	472	45.38
3. Eliminate stagnant water^c^				
No	234(22.50)	296(28.46)	530	50.96
Yes	185(17.79)	325(31.25)	510	49.04
4. Sleep under a mosquito net^b^				
No	51(4.90)	155(14.90)	206	19.81
Yes	368(35.38)	466(44.81)	834	80.19
5. Other (specify): Don’t know and clean up the living environment				
No	417(40.10)	618(59.42)	1035	99.52
Yes	2(0.19)	3(0.29)	5	0.48
6. Do nothing				
No	399(38.37)	567(54.52)	966	92.88
Yes	20(1.92)	54(5.19)	74	7.12
If you think you have dengue fever and you have symptoms, what do you do?				
1. Consult a doctor				
No	8(0.77)	7(0.67)	15	1.44
Yes	411(39.52)	614(59.04)	1025	98.56
2. Self-medication				
No	410(39.42)	617(59.33)	1027	98.75
Yes	9(0.87)	4(0.38)	13	1.25
3. Use of plants^e^				
No	375(36.06)	604(58.08)	979	94.13
Yes	44(4.23)	17(1.63)	61	5.87
4. Consult a traditional healer				
No	416(40)	620(59.62)	1036	99.62
Yes	3(.29)	1(0.10)	4	0.38
5. Prayer				
No	414(39.81)	604(58.08)	1018	97.88
Yes	5(0.48)	17(1.63)	22	2.12
6. Other (specify)				
No	418(40.19)	621(59.71)	1039	99.90
Yes	1(0.10)	0(0.0)	0	0.10
7. Do nothing^d^				
No	418(40.19)	615(59.13)	1033	99.33
Yes	1(0?10)	6(0.58)	7	0.67
If you suspect someone has dengue fever, what do you do?				
1. Advise them to go to a health Centre	411 (39.52)	615 (59.13)	1026	98.65
2. Approach them to go to a traditional practitioner	1 (0.10)	0	1	0.10
3. Approach them to start self-medicating	4 (0.38)	0 (0.00)	4	0.38
4. Do Nothing	3 (0.29)	6 (0.58)	9	0.87

This table presents self-reported attitudes against dengue fever and intended health-seeking behaviors in case of suspected illness, stratified by place of residence (rural and urban) in the national KAP survey conducted in Burkina Faso.

^**a,b,c,d,e**^ Differences between rural and urban populations were assessed using Pearson’s chi-square test. Statistically significant associations were observed for wearing long, loose clothing (χ² = 41.65; p < 0.001), sleeping under a mosquito net (χ² = 24.96; p < 0.001), eliminating stagnant water (χ² = 6.38; p = 0.011), doing nothing in response to dengue prevention (χ² = 5.25; p = 0.022), and use of medicinal plants in case of suspected dengue fever (χ² = 25.92; p < 0.001). Other comparisons were not statistically significant (p > 0.05).

When experiencing symptoms suggestive of dengue, 98.56% of respondents reported that they would consult a doctor. Very few indicated self-medication (1.25%), use of medicinal plants (5.87%), consultation of a traditional healer (0.38%), prayer (2.12%), or doing nothing (0.67%).

If they suspected someone else had dengue, 98.65% stated that they would advise the person to go to a health centre. Only small proportions reported advising traditional treatment (0.10%), self-medication (0.38%), or doing nothing (0.87%).

### Household practices towards Dengue prevention and treatment

Participants’ practices for dengue prevention and treatment are recorded in Table 5 below.

**Table 5:** Practices towards dengue fever.

Dengue practices	Rural (n, %)	Urban (n, %)	Total	Percentage
**Do you sleep under a mosquito net all the time?**				
No	126(12.12)	211(20.29)	337	32.40
Yes	293(28.17)	410(39.42)	703	67.60
**Does your household have mosquito netting on the windows?** ^**a**^				
No	410(39.42)	542(52.12)	952	91.54
Yes	9(0.87)	79(7.60)	88	8.46
**Does your household have mosquito netting on the door of the main house?** ^**b**^				
No	413(39.71)	589(56.63)	1002	96.35
Yes	6(0.58)	32(3.08)	38	3.65
**Other methods used to control mosquitoes:**				
1. Mosquito repellent spirals and sprays^c^				
No	153(14.71)	28(2.69)	181	17.40
Yes	266(25.58)	593(57.02)	859	82.60
2. Use of repellent herbs or plants^d^				
No	273(26.25)	579(55.67)	852	81.92
Yes	146(14.04)	42(4.04)	188	18.08
3. Other (please specify):none, mosquito net, environmental hygiene, don’t know, property, clean up your living environment, mosquito repellent, anti-mosquito ointment, turn the fan on all night, close the windows of houses every evening, avoid storing stagnant water, clean up your surroundings				
No	387(37.21)	603(57.98)	990	95.19
Yes	32(3.08)	18(1.73)	50	4.81
**What medication do you use if you notice signs of dengue fever?** ^**e**^				
1. Modern medicine	414(39.81)	610(58.65)	1024	98.46
2. Traditional medicine	2(0.19)	10(0.96)	12	1.15
3. Other (please specify): Don’t know	3(0.29)	1(0.10)	4	0.38

This table presents household-level preventive practices against dengue fever, including mosquito net use, structural protection measures, and mosquito control methods, as well as treatment choices in case of suspected dengue fever, stratified by place of residence (rural and urban) in the national KAP survey conducted in Burkina Faso.

a,b,c,d,e Differences between rural and urban households were assessed using Pearson’s chi-square test. Significant associations were found for household mosquito netting on windows (p < 0.001), mosquito netting on doors (p < 0.001), use of mosquito repellent spirals and sprays (χ² ≈ 118.7; p < 0.001), use of repellent herbs or plants (χ² ≈ 59.8; p < 0.001), and medication choice for suspected dengue fever (p = 0.008). No statistically significant difference was observed for other mosquito control practices (p = 0.08).

Overall, 67.60% of respondents reported sleeping under a mosquito net all the time, while 32.40% did not. Only 8.46% of households had mosquito netting on windows and 3.65% had netting on doors.

Most respondents (82.60%) reported using mosquito repellent spirals or sprays to control mosquitoes, whereas 18.08% used repellent herbs or plants and 4.81% reported other methods such as environmental hygiene or eliminating stagnant water.

Regarding treatment practices, 98.46% stated that they use modern medicine when they notice signs of dengue fever, while 1.15% reported using traditional medicine and 0.38% did not know.

## Discussion

### Socio-demographic characteristics and spatial distribution of study participants across rural and urban settings in Burkina Faso

This nationwide survey achieved a balanced representation of rural and urban populations across all thirteen regions of Burkina Faso, allowing meaningful comparisons of dengue-related knowledge, attitudes, and practices between residential settings. The study population was predominantly composed of adults aged 25–54 years, with a nearly equal distribution between males and females. Marked socio-demographic disparities were observed between rural and urban participants, particularly regarding educational level and occupation.

More than half of respondents had no formal education, especially in rural areas, whereas higher educational attainment was more frequent in urban settings. Farming predominated in rural communities, while urban participants were more engaged in trade and public or private employment sectors. Similar results on the sex ratio and age range were obtained in other KAP studies among patients attending Health Facilities in Lagos, Nigeria and on health care workers in Somalia during a cross-sectional study [13,14]. These disparities may influence access to health information and the adoption of dengue prevention measures. Lower educational attainment and predominantly outdoor occupations in rural areas may increase vulnerability to mosquito exposure and limit understanding of dengue transmission and prevention. The findings therefore highlight the importance of tailoring dengue awareness strategies according to socio-demographic and geographic contexts.

### Dengue awareness, knowledge levels, and sources of information among rural and urban populations in Burkina Faso

The study revealed moderate overall awareness of dengue fever, with important differences between urban and rural populations. Urban residents consistently demonstrated better knowledge regarding dengue transmission, recurrence, and prevention compared with rural populations.

Approximately two-thirds of respondents had heard about dengue fever, although awareness remained substantially lower in rural areas. Similar urban–rural disparities have been reported in other African and Asian settings [15,16]. These differences may reflect unequal access to health information, media exposure, and healthcare services. Rural populations often have fewer opportunities to access structured health communication campaigns.

The media and healthcare workers emerged as the main sources of dengue-related information, highlighting their central role in disseminating prevention messages. Comparable findings have been reported in previous studies conducted in dengue-endemic countries, where television, radio, and healthcare professionals constitute the primary communication channels during outbreaks [17,18].

S3 Fig extends this analysis by showing the different combinations of information sources reported by respondents. The most frequently reported combination is “health workers + media” (25.12%), which confirms the complementary nature of institutional information and media dissemination. This was followed by “friends + media” (15.88%), “health workers + friends” (12.13%), and “health workers + friends + media” (8.66%), reflecting the importance of informal exchanges as a relay for official campaigns. Certain combinations including four sources are very poorly represented, as are those involving neighbours or the “other” category, suggesting a selective and hierarchical use of information channels. Overall, more than half of the participants had been exposed to at least two sources of information. The frequent combination of multiple information sources observed in this study may contribute to better knowledge acquisition and reinforcement of preventive behaviors [19,20]. These findings highlight the strategic role of mass media and health workers in dengue prevention in Burkina Faso, while emphasizing the need to strengthen community networks and develop integrated, multi-source communication strategies to improve awareness and equitable access to health information, particularly in rural areas. Despite moderate awareness, important misconceptions persisted. Only a minority correctly identified dengue as a viral infectious disease, and many respondents remained uncertain regarding treatment availability, vaccination, and the possibility of reinfection. These findings are consistent with previous studies in sub-Saharan Africa linking limited formal education and insufficient health communication to poor dengue knowledge. [13].

While 88.27%, the vast majority of respondents correctly acknowledged that dengue can affect everyone with a higher proportion of correct responses in urban areas (53.37%) than in rural areas (34.90%), misconceptions such as dengue affecting only children or the poor were still present in a minority, indicating relatively good awareness on disease susceptibility. This reinforces the importance of targeted public education to address persistent myths [21]. Knowledge about treatment and prevention was also inadequate. Only 22.5% believed there is a drug to treat dengue, with urban participants (16.54%) being more likely to say yes than rural participants (5.96%), while 46.54% did not know. Similarly, more than half (56.60%) were unaware of the existence of a vaccine, reflecting a missed opportunity to promote immunization, particularly in high-risk areas. This uncertainty reflects either a lack of accurate information or confusion between supportive care and curative treatment. A majority of participants (56.35%) correctly stated that dengue can occur more than once. This awareness was higher among urban respondents (30.58%) compared to rural ones (25.77%). However, over one-third (38.56%) still answered “don’t know,” indicating the need to strengthen knowledge about disease recurrence and immunity. These findings demonstrate that urban populations generally have greater knowledge about dengue fever than rural populations, particularly regarding transmission, reinfection, and treatment. However, misinformation and lack of awareness remain widespread in both settings. Targeted health education campaign-especially in rural are essential to improve understanding of dengue prevention and control.

### Attitudes and preventive practices toward dengue fever and associated health-seeking behaviors in rural and urban populations of Burkina Faso

The study demonstrated generally favorable health-seeking attitudes toward dengue fever, with most participants reporting that they would seek care at health facilities in case of suspected illness. However, preventive practices differed substantially between rural and urban populations. Mosquito net use was the most commonly reported preventive measure, reflecting the long-standing impact of malaria prevention programs in Burkina Faso. While mosquito nets remain an important tool for protection against mosquito-borne diseases, dengue prevention requires additional strategies because *Aedes* mosquitoes, the primary vectors of dengue, are predominantly active during the daytime, whereas Anopheles mosquitoes, which transmit malaria, mainly bite at night. Urban residents were more likely to adopt environmental and structural preventive measures, such as eliminating stagnant water and using repellents, whereas rural populations more frequently relied on protective clothing and plant-based repellents. Similar urban–rural differences in dengue prevention practices have been described in other endemic settings [22–24]. The overwhelming preference for consulting healthcare professionals rather than relying on self-medication or traditional healers represents an encouraging finding for dengue control efforts. This pattern suggests a generally positive perception of formal healthcare services and may facilitate early diagnosis and case management. Nevertheless, some preventive measures, particularly environmental management practices, remained insufficiently adopted in both settings. These findings emphasize the need for integrated dengue prevention strategies combining environmental control, personal protection, and strengthened community engagement. Public health interventions should also capitalize on the widespread use of mosquito nets as an entry point for broader dengue prevention messaging.

### Household-level dengue prevention practices and treatment-seeking behaviors: urban–rural differentials in Burkina Faso

This study identified important differences in household-level dengue prevention strategies between rural and urban communities. Urban households generally had greater access to structural and chemical prevention tools, while rural households relied more heavily on traditional and personal protective measures.

Mosquito net use remained common overall, although structural barriers such as window and door screens were significantly more frequent in urban households. Likewise, the use of mosquito repellents and sprays was substantially higher in urban settings, likely reflecting better economic access and market availability [25–27]. In contrast, rural households more commonly used repellent plants and herbs, consistent with traditional practices reported in other African settings [28].

Environmental control measures were less frequently reported overall, suggesting limited community engagement in source reduction activities. Similar findings have been documented in other dengue-endemic regions, where household prevention efforts often focus more on personal protection than environmental management [28].

Treatment-seeking practices were largely dominated by the use of modern medicine across both rural and urban populations. This favorable behavior may contribute positively to dengue surveillance and management efforts. However, the persistence of limited traditional medicine use indicates that culturally adapted health education remains necessary.

Taken together, these findings demonstrate that dengue prevention practices are strongly influenced by socio-economic conditions, access to resources, and local cultural practices. Tailored public health interventions addressing these contextual differences are therefore essential to improve dengue prevention and control in Burkina Faso.[3,28,29].

### Limitations of the study

This study has several limitations that should be considered when interpreting the findings. First, the cross-sectional design limits the ability to infer causal relationships between knowledge, attitudes, and practices regarding dengue fever, as data were collected at a single point in time.

Second, the use of self-reported data may introduce recall bias and social desirability bias, particularly for preventive practices such as mosquito net use and health-seeking behavior, which may have been overreported by participants.

Third, although the study assessed knowledge, attitudes, and practices, the measurement of attitudes was limited. The questionnaire mainly captured behavioral intentions and preventive actions rather than psychological constructs. No Likert-scale-based measures (e.g., perceived severity, perceived susceptibility, or perceived benefits of prevention) were included, which limits the ability to fully characterize the attitudinal dimension of dengue-related perceptions.

Fourth, the reliance on household heads or their representatives as respondents may have introduced selection bias, as their responses may not fully reflect the knowledge and practices of all household members.

## Conclusion

This national study highlights important gaps in knowledge and prevention practices regarding dengue fever in Burkina Faso, with clear urban–rural disparities. Overall awareness remains moderate, with better knowledge and preventive behaviors observed in urban settings compared to rural areas.

These findings emphasize the need for targeted and context-specific health education strategies, particularly in rural communities, to improve dengue awareness and prevention. Strengthening integrated communication approaches that combine mass media, community health workers, and existing malaria control platforms could enhance the effectiveness of dengue prevention efforts.

Overall, the study provides evidence to support the development of more equitable and comprehensive national strategies for dengue control in Burkina Faso.

## Supporting information

S1 Data Household survey data. KAP Survey. The dataset contains anonymized participant-level data collected during the nationwide household survey, including socio-demographic characteristics and responses related to knowledge, attitudes, and practices regarding dengue fever in urban and rural study sites across the 13 health regions of Burkina Faso.
(XLSX)

S1 Fig Geographic distribution of the study sites in Burkina Faso. Administrative boundaries were obtained from the GADM database (https://gadm.org/download_country.html) and processed using DIVA-GIS/QGIS software. Study site locations were added by the authors using field-collected geographic coordinates. The map was generated by the authors using openly accessible geographic data under the GADM license terms (https://gadm.org/license.html).
(TIF)

S2 Fig Regional distribution of survey participants across urban and rural study sites in Burkina Faso. The figure presents the distribution of participants enrolled in the national KAP survey according to health regions and residence setting (urban versus rural), illustrating the nationwide coverage and balanced representation of the study population.
(TIF)

S3 Fig Distribution of information sources on dengue fever among participants in the national KAP survey. The figure illustrates the main channels through which respondents received information about dengue fever, including media, health workers, friends, neighbours, and other sources, as well as the combinations of these information channels reported during the survey.
(TIF)
