# Supplementary material for: Knowledge, Attitudes and Practices (KAP) on Dengue Fever in Burkina Faso: Findings from the national household survey in Burkina Faso
Source: PLoS Negl Trop Dis. 2026 Aug 31;20(8):e0014027. doi: 10.1371/journal.pntd.0014027 (PMC13552940; doi:10.1371/journal.pntd.0014027)
Supplement: Supplementary file 1 [file pntd.0014027.s006.docx]

NTD-D-26-00270
Knowledge, Attitudes and Practices (KAP) on Dengue Fever in Burkina Faso: findings from the national household survey in Burkina Faso
PLOS Neglected Tropical Diseases

**Responses to Reviewer's Questions**

**Reviewer #1:** The objectives of the study is clearly articulated. The study design is appropriate to address the stated objectives. The population is clearly described and appropriate for the hypothesis being tested. The sample size is sufficient to ensure adequate power to address the hypothesis being tested. Correct statistical analysis was used to support conclusions. There are no concerns about ethical or regulatory requirements being met. They might have included a "data analysis plan" instead of only mentioning Stata. It would have been nice if they explaiuned the choice of statistical analysis they would perform.

**Answer to Reviewer 1:**

Thank you for this important comment. We agree that a clearer description of the data analysis strategy strengthens the methodological rigor of the manuscript. We have therefore expanded the “Data Management and Analysis” section to provide a more detailed statistical analysis plan.

**Reviewer #2:**

-Are the objectives of the study clearly articulated with a clear testable hypothesis stated?

Yes, the objectives are clearly stated, but there are some concerns outlined below:

1. Introduction
Line 64-65: ‘ In 2023, dengue fever, has resurfaced in several endemic areas around the world, particularly in Burkina Faso…’.

This needs some clarity. The word ‘resurfaced’ suggests that there was no current endemicity before 2023. Is this what the authors want to say?

**Answer to Reviewer 2:**

Thank you for this insightful comment. We agree that the term “resurfaced” may incorrectly imply that dengue transmission had disappeared before 2023. Our intention was rather to emphasize the marked increase in dengue activity and outbreaks observed during 2023 in Burkina Faso and other endemic regions.

To improve clarity, we have revised the sentence as follows:

“In 2023, dengue fever continued to be a major public health challenge worldwide, with a substantial increase in reported cases in several endemic regions, including Burkina Faso, a country already facing a difficult security situation.”

This modification better reflects the ongoing endemicity of dengue while highlighting the intensified outbreak situation observed in 2023

2. References
The authors should recheck all references. Some of these do not support the claims that are being made. Examples are shown below:

Reference 8 in lines 77-79 asserts that people in Burkina Faso prefer alternative medicine for the treatment of dengue, despite ministry efforts.
The quoted paper is about the Prevalence and factors associated with self-medication in rheumatology.

a. There is no mention of dengue in the paper since this is not a rheumatologic disease
b. The study makes no mention of people in Burkina Faso's preference for alternative medicine for dengue
d. No mention of Burkina Faso ministry efforts

Reference 9 in the next lines similarly supports an unrelated assertion: the paper studied Phytopharmaceutical practices of traditional health practitioners in Burkina Faso, and makes no mention of a ‘confusion between dengue and malaria.’ as noted by the authors

The authors must recheck all references and ensure that they are providing accurate evidence in the literature to support their claims.

**Answer to Reviewer 2:**

Thank you for this careful observation. We acknowledge that some references cited in the Introduction were not appropriately aligned with the statements they were intended to support. We appreciate the reviewer for identifying these inconsistencies.

Specifically:

- Reference 8 did not provide evidence regarding the use of alternative medicine for dengue fever in Burkina Faso, nor did it discuss Ministry of Health efforts related to dengue control.
- Reference 9 similarly did not support the statement regarding confusion between dengue and malaria.

Following the reviewer’s comment, we thoroughly rechecked all references cited throughout the manuscript to ensure that each citation accurately supports the corresponding statement. The inappropriate references have been removed and replaced with more relevant literature addressing dengue-related self-medication practices, traditional treatment use, and diagnostic confusion between dengue and malaria in endemic settings.

In addition, the related sentences in the Introduction were revised for greater accuracy and consistency with the cited evidence.

3. methods
This is an important study, but the methods will benefit from some strengthening.

a. The study population needs more clarity. The authors note that they used the enumeration area of the GPHC.

First, the authors need to clearly tell the reader (especially people not familiar with Burkina Faso) what this area means…is it a community? A combination of communities clustered together? a district?

Same in line 144: ‘A total of 1,568 participants were enrolled in the study across 26 sites nationwide.’ What constitutes an area/site in this study?

-sampling

At what point did recruitment come to an end in their ‘site’? Is this when they attain a certain number of households or when all households within the area are surveyed? The authors need to be clearer about this!


-Eligibility

The authors noted that they used an eligibility criterion to sample households, but nothing else is said about this criterion. What did it entail? How many households did not meet this? How many households did not? How many households refused?

-Data collection tool

Line 120-122: ‘Data were collected using a previously validated questionnaire, developed based on a review of published KAP studies and questionnaires provided by their authors’

The authors need to rework this section.
a. While it is good to use a previously validated tool, how did they adapt to suit their context?
b. Accurate references of the said tool are important to verify the appropriateness of this study and to make sure that this study is reproducible.


-Were correct statistical analysis used to support conclusions?

Yes, however, the authors need to relook at the attitudes section. Currently, the section partly reads like it belongs in the knowledge and practice sections (e.g., personal protection practices). This section would be better off using something like a Likert scale, which is better at measuring feelings/tendencies that make up the attitude component (e.g., do participants consider dengue serious? Do they view health-seeking as important? etc. Was this done? If not, it should be mentioned in the limitations section, because the current section does not measure attitudes fully.

-Are there concerns about ethical or regulatory requirements being met?

The authors indicate that the study received ethical clearance, but failed to relay important information concerning their processes

a. Did they obtain oral or written consent prior to data collection? how did they do this?
b. What do they do when a potential participant is unable to read and write? Did they use witnesses?
c. Was there any compensation for participation

**Answer to Reviewer 2:**

Thank you very much for these detailed and constructive comments. We sincerely appreciate the reviewer’s careful evaluation of the manuscript and the valuable suggestions provided to strengthen the methodological rigor and clarity of the study.

All comments and recommendations have been carefully considered and have been fully addressed in the revised version of the manuscript. In particular, we have:

- clarified the description of the study population, enumeration areas, and study sites to improve understanding for an international readership;
- provided additional details on the sampling strategy, household selection procedures, eligibility criteria, replacement procedures, and participant recruitment endpoints;
- strengthened the description of the questionnaire development process by explaining the adaptation of previously validated KAP tools to the Burkina Faso context and by adding the appropriate references;
- **Measurement of attitudes**: We thank the reviewer for this insightful observation. We acknowledge that some variables currently presented under the “attitudes” section overlap with preventive practices and health-seeking behaviors. The questionnaire primarily focused on behavioral intentions and preventive responses rather than on psychometric assessment of attitudes using Likert-scale items.

No formal Likert-scale attitude assessment (e.g., perceived severity, perceived susceptibility, or perception of healthcare importance) was included in the original survey instrument. Following the reviewer’s recommendation, we have acknowledged this limitation in the Discussion/Limitations section and clarified that the attitude component may not fully capture the psychological dimensions of attitudes toward dengue fever.

- expand the statistical analysis section to improve transparency regarding analytical methods and interpretation;
- provide further clarification on ethical procedures, including the informed consent process for literate and non-literate participants, use of witnesses where applicable, and the absence of financial compensation.

In addition, several sections of the manuscript, including the Introduction, Methods, Results, Discussion, and Ethics Statement, have been reformulated and strengthened to improve scientific accuracy, coherence, and reproducibility.

**Results**

**Reviewer #1:** The analytical approach appears to have been conducted as intended; however, a more detailed description of the analysis plan would strengthen transparency, beyond noting the use of Stata. The results are presented comprehensively, and the figures and tables are clear and informative. It would be helpful to include the inferential statistical findings within the Results section rather than reserving them for the Discussion. Additionally, the use of subheadings in the Results section and the Discussion is recommended to improve readability and better highlight the key findings.

**Answer to Reviewer 1:**

We sincerely thank Reviewer #1 for the positive evaluation of our manuscript and for the constructive suggestions aimed at improving clarity, transparency, and readability.

In response to the reviewer’s comments, we confirm that the analysis presented is fully aligned with the planned analytical approach. However, we agree that the description of the data analysis plan was initially too brief. Therefore, in the revised manuscript, we have expanded the Methods section to provide a more detailed explanation of the statistical procedures, including descriptive statistics, comparative analyses using chi-square tests, and the handling of the survey design where applicable.

Regarding the presentation of results, we fully agree with the recommendation to improve the structure and clarity of the Results section. Accordingly, inferential statistical findings (including chi-square tests and p-values) have now been systematically integrated into the Results section rather than being discussed only in the Discussion. This change enhances transparency and allows readers to directly interpret statistical associations alongside descriptive findings.

We also concurred with the suggestion to improve readability through better structuring. In the revised manuscript, we have introduced clearer subheadings in both the Results and Discussion sections to better organize the content and highlight the main findings, including knowledge, attitudes, and practices separately, as well as urban–rural comparisons.

Finally, we confirm that all tables and figures have been carefully reviewed to ensure clarity, consistency, and adequate resolution for publication. Minor formatting adjustments have also been made where necessary to improve readability.

**Reviewer #2: -**Does the analysis presented match the analysis plan?
**Answer to Reviewer 2:**

We sincerely thank Reviewer #2 for the careful review of our manuscript and for the constructive comments aimed at strengthening the methodological transparency and clarity of presentation.

In response to the reviewer’s comments, we acknowledge that the original manuscript did not explicitly include a clearly defined statistical analysis plan. This has now been corrected in the revised version.

The Methods section has been substantially expanded to include a detailed data analysis plan, specifying the use of descriptive statistics (frequencies and percentages), inferential analyses using chi-square tests for group comparisons. This revision improves transparency and aligns the manuscript with standard reporting requirements for cross-sectional surveys.

Regarding the Results section, we agree that some parts required reorganization for clarity. The manuscript has therefore been revised to improve structure and readability, including clearer separation of KAP domains and improved presentation of key findings.

With respect to Figure 1 (study site map), we confirm that the map was generated by the authors using GADM and processed using DIVA-GIS software. In the revised manuscript, we have now explicitly stated the source of the geographic data and the software used for map construction to ensure proper attribution and reproducibility.

**Conclusions**

**Reviewer #1:** The conclusions are supported by the data presented. However, the limitations of the analysis are not clearly described and should be more explicitly addressed. The authors effectively contextualize their findings within the current literature to highlight key points. Clarifying the public health relevance of these findings for dengue control would further strengthen the manuscript.

**Answer to Reviewer** #**1:**

We thank Reviewer #1 for the positive assessment of our manuscript and for the helpful suggestions to improve clarity and public health relevance.

In response to the comments, we have revised the manuscript to more explicitly describe the study limitations. A dedicated section has now been added in the Discussion, highlighting key limitations including the cross-sectional design, self-reported data, potential recall and social desirability bias, and the limited measurement of attitudes due to the absence of Likert-scale assessment.

We have also strengthened the Discussion to better emphasize the public health implications of the findings. The revised version now more clearly highlights how urban–rural differences in knowledge and practices can guide targeted health education and integrated vector control strategies, particularly by leveraging existing malaria prevention platforms.

We thank the reviewer for these constructive comments, which have helped improve the clarity and relevance of the manuscript.

However, I would like to point out that the introduction has been revised and shortened as suggested by the peer reviewer, without omitting any of the relevant points raised by the reviewer

**Reviewer #2:** -Are the conclusions supported by the data presented?
Yes, a few concerns are listed below:
2. The conclusion section is currently wordy. These should include a general statement of the key finding of the study and its implication, not a repetition of the results.

**Answer to Reviewer** #**2:**

We sincerely thank Reviewer #2 for the careful review of our manuscript and for the constructive suggestions aimed at improving the structure and clarity of the Discussion and Conclusion sections.

In response to the reviewer’s comments, we have substantially revised the Discussion section to improve its structure and readability. The revised version now begins with a clear summary of the main findings of the study, followed by a more structured comparison with relevant literature. In addition, we have carefully reviewed the Introduction and Discussion sections to avoid repetition of content and ensure that overlapping statements have been removed or rephrased to improve conciseness and coherence.

Regarding the Conclusion section, we agree that it was previously too detailed and partly repetitive of the Results. It has now been rewritten to provide a more concise synthesis of the key findings and their public health implications, focusing on the main message and actionable recommendations rather than restating detailed results.

**Editorial and Data Presentation Modifications?**

**Reviewer #1:** I recommend minor revision because the text is lacking a little readability. No need for major analysis ort modifications. As mentioned earlier: it would be helpful to have a more detailed description of the analysis plan would strengthen transparency, beyond noting the use of Stata in the methods section. It would be helpful to include the inferential statistical findings within the Results section rather than reserving them for the Discussion. Additionally, the use of subheadings in the Results section and the Discussion is recommended to improve readability and better highlight the key findings.

**Answer to Reviewer** #**1:**

We sincerely thank Reviewer #1 for the positive overall assessment of our manuscript and the recommendation for minor revision. We appreciate the constructive suggestions aimed at improving readability and transparency.

In response to the comments, we have strengthened the Methods section by providing a more detailed description of the data analysis plan. We have also revised the Results section to include inferential statistical findings directly within the results presentation, rather than limiting them to the Discussion.

In addition, we have reorganized both the Results and Discussion sections by introducing clearer subheadings to improve structure, readability, and highlight key findings more effectively.

Reviewer #2: (No Response)

**Summary and General Comments**
**Reviewer #1:** The study addresses an important topic, given the magnitude of dengue in the country and across the African continent, as well as the limited number of publications on knowledge, attitudes, and practices (KAP) related to dengue control. Although the data were collected nearly four years ago, the topic remains highly relevant. To our knowledge, there are still no publications indexed in PubMed addressing this specific issue at the time of this review.

**Answer to Reviewer** #**1:**

We sincerely thank Reviewer #1 for this positive and encouraging assessment of our manuscript and for highlighting the relevance of the study topic in the context of dengue epidemiology in Africa.

We greatly appreciate these constructive comments, which reinforce the scientific importance and contribution of our work, and we are pleased that the study is considered suitable for publication with minor revision.

**Reviewer #2:** While this seems to be an important study, it will benefit from some strengthening; the authors need to make revisions to the methods section, describe their ethical processes better and make sure that the references they use support their claims.

**Answer to Reviewer** #**2:**

We sincerely thank Reviewer #2 for the careful review of our manuscript and for recognizing the importance of this study.

In response, we have substantially revised the Methods section to improve clarity, particularly regarding the study population, sampling approach, data collection procedures, and analysis plan. We have also strengthened the description of ethical procedures, including consent processes and field implementation details.

In addition, we have carefully reviewed all references to ensure that they appropriately support the statements made in the manuscript, and we have corrected or replaced any citations that were not fully aligned with the claims.

We thank the reviewer for these valuable suggestions, which have helped improve the methodological rigor and overall quality of the manuscript.
