# Supplementary material for: Knowledge, Attitudes and Practices (KAP) on Dengue Fever in Burkina Faso: Findings from the national household survey in Burkina Faso
Source: PLoS Negl Trop Dis. 2026 Aug 31;20(8):e0014027. doi: 10.1371/journal.pntd.0014027 (PMC13552940; doi:10.1371/journal.pntd.0014027)
Supplement: Supplementary file 2 [file pntd.0014027.s007.docx]

**Reviewers' comments:**

Reviewer's Responses to Questions

**Key Review Criteria Required for Acceptance?**
As you describe the new analyses required for acceptance, please consider the following:
**Methods**
-Are the objectives of the study clearly articulated with a clear testable hypothesis stated?
-Is the study design appropriate to address the stated objectives?
-Is the population clearly described and appropriate for the hypothesis being tested?
-Is the sample size sufficient to ensure adequate power to address the hypothesis being tested?
-Were correct statistical analysis used to support conclusions?
-Are there concerns about ethical or regulatory requirements being met?

Reviewer #1: The study objectives are clearly articulated. The study design is appropriate for addressing the stated objectives. The study population is well characterized and appropriate for the hypothesis under investigation. The sample size appears adequate to ensure sufficient statistical power. The statistical analyses employed are appropriate and support the conclusions drawn. There are no apparent concerns regarding adherence to ethical or regulatory requirements.

**Answer:**

We sincerely thank the reviewer for the careful evaluation of our manuscript and for the positive assessment of the study design, objectives, population characteristics, sample size, statistical analyses, and ethical considerations.

We appreciate the reviewer’s confirmation that the study objectives are clearly articulated, that the methodological approach is appropriate, and that the analyses adequately support our conclusions. We have taken note of these comments and have further reviewed the manuscript to ensure clarity, consistency, and compliance with the journal requirements.

Thank you again for your valuable time and constructive evaluation of our work.

Reviewer #2: Yes the objectives are clearly stated, but while the authors made some improvements to the methods section, some issues persist with other aspects of the methods section:
1. Data collection tools and questionnaire adaptation (Line 228): There seems to be a mismatch between the title and the subsequent lines (229-243). Aside the questionnaire they describe, what other 'tools' were used as indicated in the title?

**Answer:**

Thank you for this valuable comment. We agree that the previous title was not fully consistent with the content of this section. The study used a structured questionnaire as the primary and only data collection tool. The term "tools" was therefore misleading and could suggest the use of additional instruments.

To address this concern, we have revised the title of this section from **"Data collection tools and questionnaire adaptation"** to **"** **Questionnaire development, adaptation, and data collection"**. We have also reviewed the section to clarify that data collection was conducted exclusively through a structured KAP questionnaire administered during face-to-face interviews.

The revised section now provides clearer information on the development, adaptation, validation process, and administration of the questionnaire.

2. While the authors mentioned that they pretested their questionnaire, few details are given: where was this pretesting done? why did they choose this location to pretest? what was the number of sites/households where pretesting was done? Which aspects of the tool did they alter/or not, after pretesting?

**Answer:**

Thank you for this valuable comment. We agree that more details regarding the questionnaire pretesting procedure were needed.

The questionnaire pretest was conducted after the training of the data collectors and before the start of the main survey. For this purpose, the trained investigators were divided into two groups, and each group conducted a pilot administration of the questionnaire, with each group considered as a pretest site. This approach was chosen to evaluate the feasibility of the data collection procedures under field conditions similar to those of the main survey.

The pretest aimed to assess the clarity and relevance of the questions in relation to the study objectives, the understanding of items by participants, the duration of questionnaire administration, the organization of interviews, and the feasibility of household identification and localization procedures. Feedback and observations collected during this exercise were reviewed and analyzed by the study team. Based on these findings, adjustments were made to improve the wording, sequencing, and contextual adaptation of some questions before the final version of the questionnaire was implemented.

These details have been added to the revised Methods section to better describe the questionnaire development and pretesting process.

3. Ethics statement (lines 255-267): This is an improvement on the earlier submission. The authors should add in how they compensated for the time of participants.

**Answer:**

Thank you for this valuable suggestion. We have revised the Ethics Statement to clarify how participants were compensated for their time.

The present KAP survey was nested within a larger prospective national study designed to estimate dengue seroprevalence and to follow participants for one year to determine the incidence of dengue infection in Burkina Faso. No direct financial compensation was provided for participation. However, participants benefited from free medical care throughout the follow-up period. Upon presentation of their study identification card at designated health facilities, they received free consultation and treatment for suspected dengue and malaria episodes. This arrangement was intended to facilitate participation while ensuring that participants had access to appropriate medical care during the study period.

This information has now been added to the revised Ethics Statement.

4. It is essential for the authors to include a justification for their sample size. What statistical measurements helped them calculate the required sample size needed? were they able to cover this? If not, how did they ensure that their results remained statistically significant?

**Answer:**

Thank you for this important comment. We agree that the rationale for the sample size should be clearly described. We would like to clarify that this KAP survey was conducted as an additional component of a larger nationally representative dengue seroprevalence survey. Therefore, the sample size was determined based on the requirements of the primary objective of the parent study rather than specifically for the KAP outcomes.

The parent study used a multistage stratified cluster sampling design based on the 2019 General Population and Housing Census, ensuring nationwide representation across all 13 health regions of Burkina Faso, with balanced inclusion of urban and rural settings. This sampling approach was designed to provide reliable estimates at the national level while accounting for geographic, demographic, and socioeconomic variability.

Across the 26 selected study sites, 1,601 households were identified and approached. A total of 1,568 respondents were enrolled, corresponding to a participation rate of 97.9%. This achieved sample size provided a large and diverse population for assessing dengue-related knowledge, attitudes, and practices at the national level.

Although the sample size was not calculated specifically for the KAP component, the high participation rate, nationwide coverage, and inclusion of heterogeneous populations ensured adequate precision of the estimates. Furthermore, appropriate statistical methods were applied, including consideration of the complex survey design and sampling weights, to account for the probability of selection and improve the validity and representativeness of the findings.

We have added clarification in the revised Methods section regarding the origin of the sample size and its suitability for the KAP analysis.

5. The presentation of the methods section after the results does not make the manuscript easy to follow and also misses the journal requirements

6. The manuscript is difficult to follow (recheck manuscript ordering, labelling of headings, alignments, use of abbreviations without first defining them like (i.e) in line 195, and the use of double full stops 239-240, etc.).

**Answer 5 et 6:**

Thank you for your valuable comments regarding the organization and presentation of the manuscript.

We would like to clarify that the manuscript was originally structured in the conventional order (Introduction, Materials and Methods, Results, Discussion, etc.). During the first round of revisions, however, we reorganized the manuscript by moving the **Materials and Methods** section after the **Results**, following the editor's suggestion. We therefore adopted this structure in accordance with the editorial guidance provided.

We appreciate the reviewer's concern that this organization may affect the readability of the manuscript and may not fully align with the journal's standard format. We remain fully willing to reorganize the manuscript according to the journal's final requirements and will gladly restore the conventional structure if preferred.

In addition, we have carefully revised the manuscript to improve its overall presentation. We corrected the ordering and labelling of headings, defined abbreviations at their first occurrence, corrected alignment and formatting inconsistencies, and removed typographical errors, including the double full stops previously identified.

**Results**
-Does the analysis presented match the analysis plan?
-Are the results clearly and completely presented?
-Are the figures (Tables, Images) of sufficient quality for clarity?

Reviewer #1: The description of the analysis presented was according to the plan. The results were thoroughly presented. The figures and tables were clear and useful. The authors accepted our suggestions and included subtitles, and descriptions about the statistical analysis. The reading was facilitated and the scientific quality of the paper improved significantly.

**Answer:**

We sincerely thank the reviewer for the careful evaluation of our manuscript and for the positive assessment of the results presentation, statistical analysis, and quality of the figures and tables.

We appreciate the reviewer’s acknowledgment that the analyses presented were consistent with the study plan, that the results were clearly and comprehensively reported, and that the revisions made to improve the descriptions of the statistical analyses and figure/table subtitles enhanced the readability and scientific quality of the manuscript.

We have carefully considered all comments and suggestions provided during the review process, which have contributed significantly to improving the clarity, presentation, and overall quality of the revised manuscript.

Reviewer #2: Some issues remain:
1. Presentation of tables: The authors should try to avoid tables over-lapping across different pages (table 1). They should consider editing table 1 design to improve readability.

**Answer:**

Thank you for this helpful comment. We agree that the presentation and readability of tables are important for the clarity of the manuscript. In the revised version, we have carefully reformatted table 1 to improve its layout and readability. We adjusted the table design, spacing, and formatting to avoid page breaks and ensure that the table is presented clearly without overlapping across different pages.
2. S3 Fig: The authors still fail to mention the source of this figure within their manuscript. Did they develop this figure themselves? what tool helped to do this? if they got it somewhere, then they need to indicate the source.

**Answer:**

Thank you for this important comment. We confirm that the map presented in **S3 Fig** was developed by the authors and was not directly obtained from external mapping platforms. The administrative boundaries of Burkina Faso were obtained from the openly accessible GADM database and processed using **DIVA-GIS/QGIS** software. The study site locations were added by the authors based on geographic coordinates collected during the study.

In the revised manuscript, we have updated the figure legend to clearly indicate the source of the administrative shapefile (GADM) and the software used for map generation, along with the relevant licensing information.

Source of shapefile: <https://gadm.org/download_country.html>

**Conclusions**
-Are the conclusions supported by the data presented?
-Are the limitations of analysis clearly described?
-Do the authors discuss how these data can be helpful to advance our understanding of the topic under study?
-Is public health relevance addressed?

Reviewer #1: The conclusions were supported by the data presented. The limitations of analysis were clearly described. The authors discuss how these data can be helpful to advance our understanding of the topic under study drawing from current publications to highlight key points.
The study addresses an important topic, given the magnitude of dengue in the country and across the African continent, as well as the limited number of publications on knowledge, attitudes, and practices (KAP) related to dengue control. Although the data were collected nearly four years ago, the topic remains highly relevant. To our knowledge, there are still no publications indexed in PubMed addressing this specific issue at the time of this review.

Reviewer #2: Yes, but the recommened changes to the methods and results sections will strengthen this further

**Answer:**

We sincerely thank both reviewers for their positive evaluation of the conclusions and for recognizing the relevance and public health importance of this study.

We are grateful to Reviewer #1 for acknowledging that the conclusions are supported by the data presented, that the study limitations are clearly described, and that the findings contribute to advancing the current understanding of dengue-related knowledge, attitudes, and practices in Burkina Faso. We also appreciate the recognition of the study's public health relevance and its contribution to addressing an important evidence gap in the African context.

We also thank Reviewer #2 for the constructive suggestion. In response, we have revised the **Materials and Methods** and **Results** sections to improve their clarity, organization, and completeness. We believe these revisions further strengthen the manuscript and provide stronger support for the conclusions drawn.

**Editorial and Data Presentation Modifications?**
Use this section for editorial suggestions as well as relatively minor modifications of existing data that would enhance clarity. If the only modifications needed are minor and/or editorial, you may wish to recommend “Minor Revision” or “Accept”.

Reviewer #1: Nothing to be added.

Reviewer #2: The authors need to follow the journal's structure to organise their manuscript. Currently, the headings seem to have a 'thesis style' labelling (3.1, 3.2, 3.3, 3.4, 3.5, etc.).
Additionally, the authors presented their results right after the introductions and only report their methods almost at the end of the paper. They have to check the correct order and present their work as such
The presentation is difficult to follow (recheck manuscript ordering, spacing, alignments, use of abbreviations without first defining them like (i.e) in line 195, double full stops 239-240, etc.).

**Answer:**

Thank you for your careful review and constructive comments. We have thoroughly revised the manuscript to improve its organization, presentation, and readability.

Specifically, we removed the numbered subsection headings (e.g., 3.1, 3.2, 3.3), which resembled a thesis-style format, and replaced them with headings consistent with the journal's formatting requirements.

In addition, we carefully reviewed the manuscript to correct formatting and presentation issues, including manuscript ordering, spacing, alignments, undefined abbreviations (e.g., replacing i.e. with explicit wording), punctuation errors, and other typographical inconsistencies. These revisions have substantially improved the clarity and readability of the manuscript.

**Summary and General Comments**
Use this section to provide overall comments, discuss strengths/weaknesses of the study, novelty, significance, general execution and scholarship. You may also include additional comments for the author, including concerns about dual publication, research ethics, or publication ethics. If requesting major revision, please articulate the new experiments that are needed.

Reviewer #1: No request for additional changes. The text is scholarly adequate and meets the essential scientific requirements for a publication of this scope. It contributes to advance of public health in the African continent specifically regarding dengue.

Reviewer #2: The authors have made some improvements to the manuscript but important concerns persist.

**Answer:**

Thank you for your careful evaluation of the revised manuscript and for acknowledging the improvements made. We appreciate your constructive comments, which have helped us further strengthen the quality of our work.

In response to your recommendations, we have substantially revised the manuscript by providing additional methodological details on questionnaire development and pretesting, clarifying the ethical considerations and participant compensation, explaining the origin and justification of the study sample, improving the presentation of tables and figures, updating the figure legends with the appropriate data sources, and carefully reviewing the manuscript for consistency, formatting, abbreviations, and typographical errors.

We have also revised the manuscript organization to comply with the journal's formatting requirements and have sought clarification from the Editorial Office regarding the appropriate placement of the Methods section, as the editorial instructions appear to differ from one of the review comments. We believe that these revisions have addressed the remaining concerns and have considerably improved the clarity, readability, and scientific quality of the manuscript.

We sincerely thank the reviewer for the time and effort devoted to reviewing our work.

PLOS authors have the option to publish the peer review history of their article ([what does this mean?](https://track.editorialmanager.com/CL0/https:%2F%2Fjournals.plos.org%2Fplosntds%2Fs%2Feditorial-and-peer-review-process%23loc-peer-review-history/1/010f019f836e1b55-9e887951-7991-4165-a017-287915f75ba4-000000/Aq0RM1Dwea48TPZ4LGIVuNoQBdg7AH49qmXNOA1qIns=258)). If published, this will include your full peer review and any attached files.
If you choose “no”, your identity will remain anonymous but your review may still be made public.
**Do you want your identity to be public for this peer review?** For information about this choice, including consent withdrawal, please see our [Privacy Policy](https://track.editorialmanager.com/CL0/https:%2F%2Fwww.plos.org%2Fprivacy-policy/1/010f019f836e1b55-9e887951-7991-4165-a017-287915f75ba4-000000/mrZenEP7gsArjJ3wUZMZzlXdGfnW4TOw85Ny7wpRCEs=258).

Reviewer #1: No

Reviewer #2: No

[NOTE: If reviewer comments were submitted as an attachment file, they will be attached to this email and accessible via the submission site. Please log into your account, locate the manuscript record, and check for the action link "View Attachments". If this link does not appear, there are no attachment files.]

**Figure resubmission:**

While revising your submission, we strongly recommend that you use PLOS’s NAAS tool (<https://ngplosjournals.pagemajik.ai/artanalysis>) to test your figure files. NAAS can convert your figure files to the TIFF file type and meet basic requirements (such as print size, resolution), or provide you with a report on issues that do not meet our requirements and that NAAS cannot fix.
After uploading your figures to PLOS’s NAAS tool - <https://ngplosjournals.pagemajik.ai/artanalysis>, NAAS will process the files provided and display the results in the "Uploaded Files" section of the page as the processing is complete. If the uploaded figures meet our requirements (or NAAS is able to fix the files to meet our requirements), the figure will be marked as "fixed" above. If NAAS is unable to fix the files, a red "failed" label will appear above. When NAAS has confirmed that the figure files meet our requirements, please download the file via the download option, and include these NAAS processed figure files when submitting your revised manuscript.

**Reproducibility:**

To enhance the reproducibility of your results, we recommend that authors of applicable studies deposit laboratory protocols in [protocols.io](http://protocols.io/), where a protocol can be assigned its own identifier (DOI) such that it can be cited independently in the future. Additionally, PLOS ONE offers an option to publish peer-reviewed clinical study protocols. Read more information on sharing protocols at <https://plos.org/protocols?utm_medium=editorial-email&utm_source=authorletters&utm_campaign=protocols>
