# Supplementary material for: Knowledge, Attitudes and Practices (KAP) on Dengue Fever in Burkina Faso: Findings from the national household survey in Burkina Faso
Source: PLoS Negl Trop Dis. 2026 Aug 31;20(8):e0014027. doi: 10.1371/journal.pntd.0014027 (PMC13552940; doi:10.1371/journal.pntd.0014027)
Supplement: Supplementary file 3 [file pntd.0014027.s008.docx]

PNTD-D-26-00270R2

Knowledge, Attitudes and Practices (KAP) on Dengue Fever in Burkina Faso: findings from the national household survey in Burkina Faso

PLOS Neglected Tropical Diseases

Dear Dr. OUEDRAOGO,

Thank you for submitting your manuscript to PLOS Neglected Tropical Diseases. After careful consideration, we feel that the scientific and substantive responses are entirely satisfactory but structural disorganization remains an issue.  For the sake of journal uniformity and reader accessibility, we recommend that the authors follow the prescribed manuscript structure exactly as outlined in the journal’s guidelines: Title, Authors/Affiliations, Abstract, Author Summary, Introduction, Methods, Results, Discussion, Acknowledgments, References, and Supporting Information captions. Adhering to this standard format is not just a requirement—it streamlines the review process and ensures that readers can navigate the work with ease, allowing the science to speak clearly without the distraction of unconventional organization. Therefore, we invite you to submit a revised version of the manuscript that addresses this.

Please submit your revised manuscript by Oct 02 2026 11:59PM. If you will need more time than this to complete your revisions, please reply to this message or contact the journal office at [plosntds@plos.org](mailto:plosntds@plos.org). When you're ready to submit your revision, log on to <https://www.editorialmanager.com/pntd/> and select the 'Submissions Needing Revision' folder to locate your manuscript file.

Please include the following items when submitting your revised manuscript:

* A letter that responds to each point raised by the editor and reviewer(s). You should upload this letter as a separate file labeled 'Response to Reviewers'. This file does not need to include responses to any formatting updates and technical items listed in the 'Journal Requirements' section below.

* A marked-up copy of your manuscript that highlights changes made to the original version. You should upload this as a separate file labeled 'Revised Manuscript with Track Changes'.

* An unmarked version of your revised paper without tracked changes. You should upload this as a separate file labeled 'Manuscript'

If you would like to make changes to your financial disclosure, competing interests statement, or data availability statement, please make these updates within the submission form at the time of resubmission. Guidelines for resubmitting your figure files are available below the reviewer comments at the end of this letter.

As the corresponding author, your ORCID iD is verified in the submission system and will appear in the published article. PLOS supports the use of ORCID, and we encourage all coauthors to register for an ORCID iD and use it as well. Please encourage your coauthors to verify their ORCID iD within the submission system before final acceptance, as unverified ORCID iDs will not appear in the published article. Only the individual author can complete the verification step; PLOS staff cannot verify ORCID iDs on behalf of authors.

We look forward to receiving your revised manuscript.

Kind regards,

**Additional Editor Comments (if provided)**

For the sake of journal uniformity and reader accessibility, I recommend that the authors follow the prescribed manuscript structure exactly as outlined in the journal’s guidelines: Title, Authors/Affiliations, Abstract, Author Summary, Introduction, Methods, Results, Discussion, Acknowledgments, References, and Supporting Information captions. Adhering to this standard format is not just a requirement—it streamlines the review process and ensures that readers can navigate the work with ease, allowing the science to speak clearly without the distraction of unconventional organization.

**Reviewers' comments:**

[NOTE: If reviewer comments were submitted as an attachment file, they will be attached to this email and accessible via the submission site. Please log into your account, locate the manuscript record, and check for the action link "View Attachments". If this link does not appear, there are no attachment files.]

**Figure resubmission:**

While revising your submission, we strongly recommend that you use PLOS’s NAAS tool (<https://ngplosjournals.pagemajik.ai/artanalysis>) to test your figure files. NAAS can convert your figure files to the TIFF file type and meet basic requirements (such as print size, resolution), or provide you with a report on issues that do not meet our requirements and that NAAS cannot fix.
After uploading your figures to PLOS’s NAAS tool - <https://ngplosjournals.pagemajik.ai/artanalysis>, NAAS will process the files provided and display the results in the "Uploaded Files" section of the page as the processing is complete. If the uploaded figures meet our requirements (or NAAS is able to fix the files to meet our requirements), the figure will be marked as "fixed" above. If NAAS is unable to fix the files, a red "failed" label will appear above. When NAAS has confirmed that the figure files meet our requirements, please download the file via the download option, and include these NAAS processed figure files when submitting your revised manuscript.

**Reproducibility:**

To enhance the reproducibility of your results, we recommend that authors of applicable studies deposit laboratory protocols in [protocols.io](http://protocols.io/), where a protocol can be assigned its own identifier (DOI) such that it can be cited independently in the future. Additionally, PLOS ONE offers an option to publish peer-reviewed clinical study protocols. Read more information on sharing protocols at <https://plos.org/protocols?utm_medium=editorial-email&utm_source=authorletters&utm_campaign=protocols>

**Responses:**

Dear Editor,

We sincerely appreciate the Editorial Office’s positive assessment of our scientific and substantive responses. We also acknowledge the remaining concern regarding the structural organization of the manuscript.

In response to the Editor’s recommendation, we have carefully revised and reorganized the manuscript to follow the prescribed PLOS Neglected Tropical Diseases structure exactly, namely: Title, Authors/Affiliations, Abstract, Author Summary, Introduction, Methods, Results, Discussion, Acknowledgments, References, and Supporting Information captions.

In addition, we will provide the required marked-up version with tracked changes, and a clean version of the revised manuscript upon resubmission.

We are grateful for the Editor’s guidance, which has helped us improve the organization, readability, and accessibility of the manuscript. We hope that the revised version will now meet the journal’s structural requirements and be suitable for further consideration
