# Supplementary material for: Knowledge, Attitudes and Practices (KAP) on Dengue Fever in Burkina Faso: Findings from the national household survey in Burkina Faso
Source: PLoS Negl Trop Dis. 2026 Aug 31;20(8):e0014027. doi: 10.1371/journal.pntd.0014027 (PMC13552940; doi:10.1371/journal.pntd.0014027)
Supplement: Peer review history

# Peer review: Decision Letter 0

**DOI:** 10.1371/journal.pntd.0014027.r001

**Authors:** Richard Phillips, Stephanie Seifert

17 May 2026

PNTD-D-26-00270

Knowledge, Attitudes and Practices (KAP) on Dengue Fever in Burkina Faso: findings from the national household survey in Burkina Faso

PLOS Neglected Tropical Diseases

Dear Dr. OUEDRAOGO,

Thank you for submitting your manuscript to PLOS Neglected Tropical Diseases. After careful consideration, we feel that it has merit but does not fully meet PLOS Neglected Tropical Diseases’s publication criteria as it currently stands. Therefore, we invite you to submit a revised version of the manuscript that addresses the points raised during the review process.

Please submit your revised manuscript within by Jul 16 2026 11:59PM. If you will need more time than this to complete your revisions, please reply to this message or contact the journal office at plosntds@plos.org. When you're ready to submit your revision, log on to https://www.editorialmanager.com/pntd/ and select the 'Submissions Needing Revision' folder to locate your manuscript file.

Please include the following items when submitting your revised manuscript:

* A letter that responds to each point raised by the editor and reviewer(s). You should upload this letter as a separate file labeled 'Response to Reviewers'. This file does not need to include responses to any formatting updates and technical items listed in the 'Journal Requirements' section below.

* A marked-up copy of your manuscript that highlights changes made to the original version. You should upload this as a separate file labeled 'Revised Manuscript with Track Changes'.

* An unmarked version of your revised paper without tracked changes. You should upload this as a separate file labeled 'Manuscript'.

If you would like to make changes to your financial disclosure, competing interests statement, or data availability statement, please make these updates within the submission form at the time of resubmission. Guidelines for resubmitting your figure files are available below the reviewer comments at the end of this letter.

As the corresponding author, your ORCID iD is verified in the submission system and will appear in the published article. PLOS supports the use of ORCID, and we encourage all coauthors to register for an ORCID iD and use it as well. Please encourage your coauthors to verify their ORCID iD within the submission system before final acceptance, as unverified ORCID iDs will not appear in the published article. Only the individual author can complete the verification step; PLOS staff cannot verify ORCID iDs on behalf of authors.

We look forward to receiving your revised manuscript.

Kind regards,

Richard Odame Phillips

Academic Editor

PLOS Neglected Tropical Diseases

Stephanie Seifert

Section Editor

PLOS Neglected Tropical Diseases

Shaden Kamhawi

co-Editor-in-Chief

PLOS Neglected Tropical Diseases

orcid.org/0000-0003-4304-636XX

Paul Brindley

co-Editor-in-Chief

PLOS Neglected Tropical Diseases

orcid.org/0000-0003-1765-0002

**Journal Requirements:**

If the reviewer comments include a recommendation to cite specific previously published works, please review and evaluate these publications to determine whether they are relevant and should be cited. There is no requirement to cite these works unless the editor has indicated otherwise.

1) Please ensure that the CRediT author contributions listed for every co-author are completed accurately and in full.

At this stage, the following Authors/Authors require contributions: Nicolas OUEDRAOGO. Please ensure that the full contributions of each author are acknowledged in the "Add/Edit/Remove Authors" section of our submission form.

The list of CRediT author contributions may be found here: https://journals.plos.org/plosntds/s/authorship#loc-author-contributions

2) We ask that a manuscript source file is provided at Revision. Please upload your manuscript file as a .doc, .docx, .rtf or .tex. If you are providing a .tex file, please upload it under the item type u2018LaTeX Source Fileu2019 and leave your .pdf version as the item type u2018Manuscriptu2019.

3) Your manuscript's sections are not in the correct order.  Please amend to the following order: Abstract, Introduction, Results, Discussion, and Methods

4) Thank you for including an Ethics Statement for your study. Please include:

i) A statement that formal consent was obtained (must state whether verbal/written) OR the reason consent was not obtained (e.g. anonymity). NOTE: If child participants, the statement must declare that formal consent was obtained from the parent/guardian.].

5) Please upload all main figures as separate Figure files in .tif or .eps format. For more information about how to convert and format your figure files please see our guidelines:

https://journals.plos.org/plosntds/s/figures

6) We have noticed that you have uploaded Supporting Information files, but you have not included a list of legends. Please add a full list of legends for your Supporting Information files after the references list.

7) Some material included in your submission may be copyrighted. According to PLOS’s copyright policy, authors who use figures or other material (e.g., graphics, clipart, maps) from another author or copyright holder must demonstrate or obtain permission to publish this material under the Creative Commons Attribution 4.0 International (CC BY 4.0) License used by PLOS journals. Please closely review the details of PLOS’s copyright requirements here: PLOS Licenses and Copyright. If you need to request permissions from a copyright holder, you may use PLOS's Copyright Content Permission form.

Please respond directly to this email and provide any known details concerning your material's license terms and permissions required for reuse, even if you have not yet obtained copyright permissions or are unsure of your material's copyright compatibility. Once you have responded and addressed all other outstanding technical requirements, you may resubmit your manuscript within Editorial Manager.

Potential Copyright Issues:

i) Figure 1. Please (a) provide a direct link to the base layer of the map (i.e., the country or region border shape) and ensure this is also included in the figure legend; and (b) provide a link to the terms of use / license information for the base layer image or shapefile. We cannot publish proprietary or copyrighted maps (e.g. Google Maps, Mapquest) and the terms of use for your map base layer must be compatible with our CC BY 4.0 license.

Note: if you created the map in a software program like R or ArcGIS, please locate and indicate the source of the basemap shapefile onto which data has been plotted.

If your map was obtained from a copyrighted source please amend the figure so that the base map used is from an openly available source. Alternatively, please provide explicit written permission from the copyright holder granting you the right to publish the material under our CC BY 4.0 license.

If you are unsure whether you can use a map or not, please do reach out and we will be able to help you. The following websites are good examples of where you can source open access or public domain maps:

* U.S. Geological Survey (USGS) - All maps are in the public domain. (http://www.usgs.gov)

* PlaniGlobe - All maps are published under a Creative Commons license so please cite u201cPlaniGlobe, http://www.planiglobe.com, CC BY 2.0u201d in the image credit after the caption. (http://www.planiglobe.com/?lang=enl)

* Natural Earth - All maps are public domain. (http://www.naturalearthdata.com/about/terms-of-use/).

8) In the online submission form, you indicated that The data underlying the findings of this study are available from the corresponding author upon reasonable request. All PLOS journals now require all data underlying the findings described in their manuscript to be freely available to other researchers, either

1. In a public repository

2. Within the manuscript itself

3. Uploaded as supplementary information.

This policy applies to all data except where public deposition would breach compliance with the protocol approved by your research ethics board. If your data cannot be made publicly available for ethical or legal reasons (e.g., public availability would compromise patient privacy), please explain your reasons by return email and your exemption request will be escalated to the editor for approval. Your exemption request will be handled independently and will not hold up the peer review process, but will need to be resolved should your manuscript be accepted for publication. One of the Editorial team will then be in touch if there are any issues.

9) Please provide a detailed Financial Disclosure statement. This is published with the article. It must therefore be completed in full sentences and contain the exact wording you wish to be published.

1) Please clarify all sources of financial support for your study. List the grants, grant numbers, and organizations that funded your study, including funding received from your institution. Please note that suppliers of material support, including research materials, should be recognized in the Acknowledgements section rather than in the Financial Disclosure

2) State the initials, alongside each funding source, of each author to receive each grant. For example: "This work was supported by the National Institutes of Health (####### to AM; ###### to CJ) and the National Science Foundation (###### to AM)."

3) State what role the funders took in the study. If the funders had no role in your study, please state: "The funders had no role in study design, data collection and analysis, decision to publish, or preparation of the manuscript."

4) If any authors received a salary from any of your funders, please state which authors and which funders..

If you did not receive any funding for this study, please simply state: u201cThe authors received no specific funding for this work.u201d

10) Your current Financial Disclosure states, "The author(s) received no specific funding for this work.".

However, your funding information on the submission form indicates receiving fund from World Bank through the Health Services Reinforcement Project (Projet de Renforcement des Services de Santé, PRSS)..

Please indicate by return email the full and correct funding information for your study and confirm the order in which funding contributions should appear. Please be sure to indicate whether the funders played any role in the study design, data collection and analysis, decision to publish, or preparation of the manuscript.

**Reviewers' Comments:**

Reviewer's Responses to Questions

**Key Review Criteria Required for Acceptance?**

As you describe the new analyses required for acceptance, please consider the following:

**Methods**

-Are the objectives of the study clearly articulated with a clear testable hypothesis stated?

-Is the study design appropriate to address the stated objectives?

-Is the population clearly described and appropriate for the hypothesis being tested?

-Is the sample size sufficient to ensure adequate power to address the hypothesis being tested?

-Were correct statistical analysis used to support conclusions?

-Are there concerns about ethical or regulatory requirements being met?

Reviewer #1: The objectives of the study is clearly articulated. The study design is appropriate to address the stated objectives. The population is clearly described and appropriate for the hypothesis being tested. The sample size is sufficient to ensure adequate power to address the hypothesis being tested. Correct statistical analysis was used to support conclusions. There are no concerns about ethical or regulatory requirements being met. They might have included a "data analysis plan" instead of only mentioning Stata. It would have been nice if they explaiuned thge choice of statistical analysis they would perform.

Reviewer #2: -Are the objectives of the study clearly articulated with a clear testable hypothesis stated?

Yes, the objectives are clearly stated, but there are some concerns outlined below:

1. Introduction

Line 64-65: ‘ In 2023, dengue fever, has resurfaced in several endemic areas around the world, particularly in Burkina Faso…’.

This needs some clarity. The word ‘resurfaced’ suggests that there was no current endemicity before 2023. Is this what the authors want to say?

2. References

The authors should recheck all references. Some of these do not support the claims that are being made. Examples are shown below:

Reference 8 in lines 77-79 asserts that people in Burkina Faso prefer alternative medicine for the treatment of dengue, despite ministry efforts.

The quoted paper is about the Prevalence and factors associated with self-medication in rheumatology.

a. There is no mention of dengue in the paper since this is not a rheumatologic disease

b. The study makes no mention of people in Burkina Faso's preference for alternative medicine for dengue

d. No mention of Burkina Faso ministry efforts

Reference 9 in the next lines similarly supports an unrelated assertion: the paper studied Phytopharmaceutical practices of traditional health practitioners in Burkina Faso, and makes no mention of a ‘confusion between dengue and malaria.’ as noted by the authors

The authors must recheck all references and ensure that they are providing accurate evidence in the literature to support their claims.

3. methods

This is an important study, but the methods will benefit from some strengthening.

a. The study population needs more clarity. The authors note that they used the enumeration area of the GPHC.

First, the authors need to clearly tell the reader (especially people not familiar with Burkina Faso) what this area means…is it a community? A combination of communities clustered together? a district?

Same in line 144: ‘A total of 1,568 participants were enrolled in the study across 26 sites nationwide.’ What constitutes an area/site in this study?

-sampling

At what point did recruitment come to an end in their ‘site’? Is this when they attain a certain number of households or when all households within the area are surveyed? The authors need to be clearer about this!

-Eligibility

The authors noted that they used an eligibility criterion to sample households, but nothing else is said about this criterion. What did it entail? How many households did not meet this? How many households did not? How many households refused?

-Data collection tool

Line 120-122: ‘Data were collected using a previously validated questionnaire, developed based on a review of published KAP studies and questionnaires provided by their authors’

The authors need to rework this section.

a. While it is good to use a previously validated tool, how did they adapt to suit their context?

b. Accurate references of the said tool are important to verify the appropriateness of this study and to make sure that this study is reproducible.

-Were correct statistical analysis used to support conclusions?

Yes, however, the authors need to relook at the attitudes section. Currently, the section partly reads like it belongs in the knowledge and practice sections (e.g., personal protection practices). This section would be better off using something like a Likert scale, which is better at measuring feelings/tendencies that make up the attitude component (e.g., do participants consider dengue serious? Do they view health-seeking as important?, etc. Was this done? If not, it should be mentioned in the limitations section, because the current section does not measure attitudes fully.

-Are there concerns about ethical or regulatory requirements being met?

The authors indicate that the study received ethical clearance, but failed to relay important information concerning their processes

a. Did they obtain oral or written consent prior to data collection? how did they do this?

b. What do they do when a potential participant is unable to read and write? Did they use witnesses?

c. Was there any compensation for participation

**********

**Results**

-Does the analysis presented match the analysis plan?

-Are the results clearly and completely presented?

-Are the figures (Tables, Images) of sufficient quality for clarity?

Reviewer #1: The analytical approach appears to have been conducted as intended; however, a more detailed description of the analysis plan would strengthen transparency, beyond noting the use of Stata. The results are presented comprehensively, and the figures and tables are clear and informative. It would be helpful to include the inferential statistical findings within the Results section rather than reserving them for the Discussion. Additionally, the use of subheadings in the Results section and the Discussion is recommended to improve readability and better highlight the key findings.

Reviewer #2: -Does the analysis presented match the analysis plan?

The authors failed to mention an analysis plan. There is currently no mention on the type of analysis they had intended to run in the methods section

-Are the results clearly and completely presented?

Yes, but as indicated some sections need to be reworked

-Are the figures (Tables, Images) of sufficient quality for clarity?

Yes, but where did the authors get their study site map (fig. 1), Did they generate this themselves or not? the source needs to be acknowledged

**********

**Conclusions**

-Are the conclusions supported by the data presented?

-Are the limitations of analysis clearly described?

-Do the authors discuss how these data can be helpful to advance our understanding of the topic under study?

-Is public health relevance addressed?

Reviewer #1: The conclusions are supported by the data presented. However, the limitations of the analysis are not clearly described and should be more explicitly addressed. The authors effectively contextualize their findings within the current literature to highlight key points. Clarifying the public health relevance of these findings for dengue control would further strengthen the manuscript.

Reviewer #2: -Are the conclusions supported by the data presented?

Yes, a few concerns are listed below:

1. Discussion:

a. Start your discussion by summarising for the reader what your study found.

b. Then compare with other studies, etc. As you do, but

c. Read over your intro and eliminate anything that you again mention in the discussion.

2. The conclusion section is currently wordy. These should include a general statement of the key finding of the study and its implication, not a repetition of the results.

**********

**Editorial and Data Presentation Modifications?**

Use this section for editorial suggestions as well as relatively minor modifications of existing data that would enhance clarity. If the only modifications needed are minor and/or editorial, you may wish to recommend “Minor Revision” or “Accept”.

Reviewer #1: I recommend minor revision because the text is lacking a little readability. No need for major analysis ort modifications. As mentioned earlier: it would be helpful to have a more detailed description of the analysis plan would strengthen transparency, beyond noting the use of Stata in the methods section. It would be helpful to include the inferential statistical findings within the Results section rather than reserving them for the Discussion. Additionally, the use of subheadings in the Results section and the Discussion is recommended to improve readability and better highlight the key findings.

Reviewer #2: (No Response)

**********

**Summary and General Comments**

Use this section to provide overall comments, discuss strengths/weaknesses of the study, novelty, significance, general execution and scholarship. You may also include additional comments for the author, including concerns about dual publication, research ethics, or publication ethics. If requesting major revision, please articulate the new experiments that are needed.

Reviewer #1: The study addresses an important topic, given the magnitude of dengue in the country and across the African continent, as well as the limited number of publications on knowledge, attitudes, and practices (KAP) related to dengue control. Although the data were collected nearly four years ago, the topic remains highly relevant. To our knowledge, there are still no publications indexed in PubMed addressing this specific issue at the time of this review.

Reviewer #2: While this seems to be an important study, it will benefit from some strengthening; the authors need to make revisions to the methods section, describe their ethical processes better and make sure that the references they use support their claims.

**********

PLOS authors have the option to publish the peer review history of their article (what does this mean?). If published, this will include your full peer review and any attached files.

If you choose “no”, your identity will remain anonymous but your review may still be made public.

**Do you want your identity to be public for this peer review?**  For information about this choice, including consent withdrawal, please see our Privacy Policy.

Reviewer #1: No

Reviewer #2: No

**Figure resubmission:**

While revising your submission, we strongly recommend that you use PLOS’s NAAS tool (https://ngplosjournals.pagemajik.ai/artanalysis) to test your figure files. NAAS can convert your figure files to the TIFF file type and meet basic requirements (such as print size, resolution), or provide you with a report on issues that do not meet our requirements and that NAAS cannot fix.

**Reproducibility:**

To enhance the reproducibility of your results, we recommend that authors of applicable studies deposit laboratory protocols in protocols.io, where a protocol can be assigned its own identifier (DOI) such that it can be cited independently in the future. Additionally, PLOS ONE offers an option to publish peer-reviewed clinical study protocols. Read more information on sharing protocols at https://plos.org/protocols?utm_medium=editorial-email&utm_source=authorletters&utm_campaign=protocols

# Author comment: Author response to Decision Letter 1

**DOI:** 10.1371/journal.pntd.0014027.r002

2 Jun 2026

Attachment Submitted filename: Response to Reviewers.docx

# Peer review: Decision Letter 1

**DOI:** 10.1371/journal.pntd.0014027.r003

**Authors:** Richard Phillips, Stephanie Seifert

21 Jul 2026

Response to Reviewers Revised Manuscript with Track Changes

* An unmarked version of your revised paper without tracked changes. You should upload this as a separate file labeled 'Manuscript'.

Shaden Kamhawi

co-Editor-in-Chief

PLOS Neglected Tropical Diseases

orcid.org/0000-0003-4304-636XX

Paul Brindley

co-Editor-in-Chief

PLOS Neglected Tropical Diseases

orcid.org/0000-0003-1765-0002

**Additional Editor Comments (if provided):** **Reviewers' comments:**

**Key Review Criteria Required for Acceptance?**

As you describe the new analyses required for acceptance, please consider the following:

**Methods**

-Are the objectives of the study clearly articulated with a clear testable hypothesis stated?

-Is the study design appropriate to address the stated objectives?

-Is the population clearly described and appropriate for the hypothesis being tested?

-Is the sample size sufficient to ensure adequate power to address the hypothesis being tested?

-Were correct statistical analysis used to support conclusions?

-Are there concerns about ethical or regulatory requirements being met?

Reviewer #1: The study objectives are clearly articulated. The study design is appropriate for addressing the stated objectives. The study population is well characterized and appropriate for the hypothesis under investigation. The sample size appears adequate to ensure sufficient statistical power. The statistical analyses employed are appropriate and support the conclusions drawn. There are no apparent concerns regarding adherence to ethical or regulatory requirements.

Reviewer #2: Yes the objectives are clearly stated, but while the authors made some improvements to the methods section, some issues persist with other aspects of the methods section:

1. Data collection tools and questionnaire adaptation (Line 228): There seems to be a mismatch between the title and the subsequent lines (229-243). Aside the questionnaire they describe, what other 'tools' were used as indicated in the title?

2. While the authors mentioned that they pretested their questionnaire, few details are given: where was this pretesting done? why did they choose this location to pretest? what was the number of sites/households where pretesting was done? Which aspects of the tool did they alter/or not, after pretesting?

3. Ethics statement (lines 255-267): This is an improvement on the earlier submission. The authors should add in how they compensated for the time of participants

4. It is essential for the authors to include a justification for their sample size. What statistical measurements helped them calculate the required sample size needed? were they able to cover this? If not, how did they ensure that their results remained statistically significant?

5. The presentation of the methods section after the results does not make the manuscript easy to follow and also misses the journal requirements

6. The manuscipt is difficult to follow (recheck manuscript ordering, labelling of headings, alignments, use of abbreviations without first defining them like (i.e) in line 195, and the use of double full stops 239-240, etc.).

**********

**Results**

-Does the analysis presented match the analysis plan?

-Are the results clearly and completely presented?

-Are the figures (Tables, Images) of sufficient quality for clarity?

Reviewer #1: The description of the analysis presented was according to the plan. The results were thoroughly presented. The figures and tables were clear and useful. The authors accepted our suggestions and included subtitles, and descriptions about the statistical analysis. The reading was facilitated and the scientific quality of the paper improved significantly.

Reviewer #2: Some issues remain:

1. Presentation of tables: The authors should try to avoid tables over-lapping across different pages (table 1). They should consider editing table 1 design to improve readability

2. S3 Fig: The authors still fail to mention the source of this figure within their manuscript. Did they develop this figure themselves? what tool helped to do this? if they got it somewhere, then they need to indicate the source

**********

**Conclusions**

-Are the conclusions supported by the data presented?

-Are the limitations of analysis clearly described?

-Do the authors discuss how these data can be helpful to advance our understanding of the topic under study?

-Is public health relevance addressed?

Reviewer #1: The conclusions were supported by the data presented. The limitations of analysis were clearly described. The authors discuss how these data can be helpful to advance our understanding of the topic under study drawing from current publications to highlight key points.

The study addresses an important topic, given the magnitude of dengue in the country and across the African continent, as well as the limited number of publications on knowledge, attitudes, and practices (KAP) related to dengue control. Although the data were collected nearly four years ago, the topic remains highly relevant. To our knowledge, there are still no publications indexed in PubMed addressing this specific issue at the time of this review.

Reviewer #2: Yes, but the recommened changes to the methods and results secitons will strengthen this further

**********

**Editorial and Data Presentation Modifications?**

Use this section for editorial suggestions as well as relatively minor modifications of existing data that would enhance clarity. If the only modifications needed are minor and/or editorial, you may wish to recommend “Minor Revision” or “Accept”.

Reviewer #1: Nothing to be added.

Reviewer #2: The authors need to follow the journal's structure to organise their manuscript. Currently, the headings seem to have a 'thesis style' labelling (3.1, 3.2, 3.3, 3.4, 3.5, etc.).

Additionally, the authors presented their results right after the introductions and only report their methods almost at the end of the paper. They have to check the correct order and present their work as such

The presentation is difficult to follow (recheck manuscript ordering, spacing, alignments, use of abbreviations without first defining them like (i.e) in line 195, double full stops 239-240, etc.).

**********

**Summary and General Comments**

Use this section to provide overall comments, discuss strengths/weaknesses of the study, novelty, significance, general execution and scholarship. You may also include additional comments for the author, including concerns about dual publication, research ethics, or publication ethics. If requesting major revision, please articulate the new experiments that are needed.

Reviewer #1: No request for additional changes. The text is scholarly adequate and meets the essential scientific requirements for a publication of this scope. It contributes to advance of public health in the African continent specifically regarding dengue.

Reviewer #2: The authors have made some improvements to the manuscript but important concerns persist.

**********

PLOS authors have the option to publish the peer review history of their article (what does this mean?). If published, this will include your full peer review and any attached files.

If you choose “no”, your identity will remain anonymous but your review may still be made public.

**Do you want your identity to be public for this peer review?**  For information about this choice, including consent withdrawal, please see our Privacy Policy.

Reviewer #1: No

Reviewer #2: No

**Figure resubmission:**

**Reproducibility:**

To enhance the reproducibility of your results, we recommend that authors of applicable studies deposit laboratory protocols in protocols.io, where a protocol can be assigned its own identifier (DOI) such that it can be cited independently in the future. Additionally, PLOS ONE offers an option to publish peer-reviewed clinical study protocols. Read more information on sharing protocols at https://plos.org/protocols?utm_medium=editorial-email&utm_source=authorletters&utm_campaign=protocols

# Author comment: Author response to Decision Letter 2

**DOI:** 10.1371/journal.pntd.0014027.r004

22 Jul 2026

Attachment Submitted filename: Response_to_Reviewers_auresp_2.docx

# Editor report: Decision Letter 2

**DOI:** 10.1371/journal.pntd.0014027.r005

**Authors:** Richard Phillips, Stephanie Seifert

3 Aug 2026

Response to Reviewers Revised Manuscript with Track Changes Manuscript

Shaden Kamhawi

co-Editor-in-Chief

PLOS Neglected Tropical Diseases

orcid.org/0000-0003-4304-636XX

Paul Brindley

co-Editor-in-Chief

PLOS Neglected Tropical Diseases

orcid.org/0000-0003-1765-0002

**Additional Editor Comments (if provided):**

**Reviewers' comments:** **Figure resubmission:**

**Reproducibility:**

To enhance the reproducibility of your results, we recommend that authors of applicable studies deposit laboratory protocols in protocols.io, where a protocol can be assigned its own identifier (DOI) such that it can be cited independently in the future. Additionally, PLOS ONE offers an option to publish peer-reviewed clinical study protocols. Read more information on sharing protocols at https://plos.org/protocols?utm_medium=editorial-email&utm_source=authorletters&utm_campaign=protocols

# Author comment: Author response to Decision Letter 3

**DOI:** 10.1371/journal.pntd.0014027.r006

10 Aug 2026

Attachment Submitted filename: Response_to_Reviewers_auresp_3.docx

# Editor report: Decision Letter 3

**DOI:** 10.1371/journal.pntd.0014027.r007

**Authors:** Richard Phillips, Stephanie Seifert

23 Aug 2026

Dear Biologiste OUEDRAOGO,

We are pleased to inform you that your manuscript 'Knowledge, Attitudes and Practices (KAP) on Dengue Fever in Burkina Faso: findings from the national household survey in Burkina Faso' has been provisionally accepted for publication in PLOS Neglected Tropical Diseases.

Before your manuscript can be formally accepted you will need to complete some formatting changes, which you will receive in a follow up email. A member of our team will be in touch with a set of requests.

Please note that your manuscript will not be scheduled for publication until you have made the required changes, so a swift response is appreciated.

IMPORTANT: The editorial review process is now complete. PLOS will only permit corrections to spelling, formatting or significant scientific errors from this point onwards. Requests for major changes, or any which affect the scientific understanding of your work, will cause delays to the publication date of your manuscript.

Should you, your institution's press office or the journal office choose to press release your paper, you will automatically be opted out of early publication. We ask that you notify us now if you or your institution is planning to press release the article. All press must be co-ordinated with PLOS.

Thank you again for supporting Open Access publishing; we are looking forward to publishing your work in PLOS Neglected Tropical Diseases.

Best regards,

Richard Odame Phillips

Academic Editor

PLOS Neglected Tropical Diseases

Stephanie Seifert

Section Editor

PLOS Neglected Tropical Diseases

Shaden Kamhawi

co-Editor-in-Chief

PLOS Neglected Tropical Diseases

orcid.org/0000-0003-4304-636XX

Paul Brindley

co-Editor-in-Chief

PLOS Neglected Tropical Diseases

orcid.org/0000-0003-1765-0002

***********************************************************

p.p1 {margin: 0.0px 0.0px 0.0px 0.0px; line-height: 16.0px; font: 14.0px Arial; color: #323333; -webkit-text-stroke: #323333}span.s1 {font-kerning: none

# Editor report: Acceptance letter

**DOI:** 10.1371/journal.pntd.0014027.r008

**Authors:** Richard Phillips, Stephanie Seifert

Dear Biologiste OUEDRAOGO,

We are delighted to inform you that your manuscript, "Knowledge, Attitudes and Practices (KAP) on Dengue Fever in Burkina Faso: findings from the national household survey in Burkina Faso," has been formally accepted for publication in PLOS Neglected Tropical Diseases.

We have now passed your article onto the PLOS Production Department who will complete the rest of the publication process. All authors will receive a confirmation email upon publication.

The corresponding author will soon be receiving a typeset proof for review, to ensure errors have not been introduced during production. Please review the PDF proof of your manuscript carefully, as this is the last chance to correct any scientific or type-setting errors. Please note that major changes, or those which affect the scientific understanding of the work, will likely cause delays to the publication date of your manuscript. Note: Proofs for Front Matter articles (Editorial, Viewpoint, Symposium, Review, etc...) are generated on a different schedule and may not be made available as quickly.

Soon after your final files are uploaded, the early version of your manuscript will be published online unless you opted out of this process. The date of the early version will be your article's publication date. The final article will be published to the same URL, and all versions of the paper will be accessible to readers.

For Research Articles, you will receive an invoice from PLOS for your publication fee after your manuscript has reached the completed accept phase. If you receive an email requesting payment before acceptance or for any other service, this may be a phishing scheme. Learn how to identify phishing emails and protect your accounts at https://explore.plos.org/phishing.

Thank you again for supporting open-access publishing; we are looking forward to publishing your work in PLOS Neglected Tropical Diseases.

Best regards,

Shaden Kamhawi

co-Editor-in-Chief

PLOS Neglected Tropical Diseases

Paul Brindley

co-Editor-in-Chief

PLOS Neglected Tropical Diseases
